# Foundations of Pediatric Lifestyle Medicine

**DOI:** 10.3390/children12030304

**Published:** 2025-02-27

**Authors:** Marina Gaínza-Lein

**Affiliations:** 1 Instituto de Pediatría, Facultad de Medicina, Universidad Austral de Chile, Independencia 631, Valdivia 5110566, Chile; marina.gainza@uach.cl; 2 Diplomado en Medicina del Estilo de Vida Infantil, Sembrando Salud, Valdivia 5110683, Chile; 3 Facultad de Medicina, Universidad de Chile, Santiago 8380453, Chile

**Keywords:** pediatrics, lifestyle medicine, pediatric lifestyle, medicine family, medicine children

## Abstract

**Background:** Lifestyle medicine utilizes therapeutic interventions to prevent, treat, and reverse chronic diseases by promoting healthy habits. While extensively studied in adults, its application in pediatrics remains underexplored. Given that many chronic diseases originate in early life, establishing the foundations of Pediatric Lifestyle Medicine is essential. **Methods**: This paper presents a comprehensive literature review and clinical insights to assess the evidence supporting Pediatric Lifestyle Medicine and provide age-appropriate, evidence-based guidelines for children and adolescents. **Results**: Pediatric Lifestyle Medicine is an evidence-based healthcare discipline focused on promoting and maintaining children’s health by encouraging healthy habits from an early age. This approach prevents chronic diseases, supports physical and emotional well-being, and fosters long-term quality of life. Findings show that applying its principles in childhood can prevent obesity, improve mental health, and aid in disease management, while also reducing the risk of adult-onset conditions and benefiting planetary health. Pediatric Lifestyle Medicine is based on six pillars: preventive nutrition, physical activity, sleep, stress management, positive social connections, and risk prevention, the last of which includes toxin avoidance and other childhood-specific risks, such as accident prevention and screen overuse. **Conclusions**: Pediatric Lifestyle Medicine provides a cost-effective, evidence-based framework for improving childhood health and preventing chronic conditions. Integrating these principles into pediatric care can foster lifelong health benefits, emphasizing the need for further research and implementation in medical education.

## 1. Introduction

Lifestyle medicine is a newly established specialty aimed at preventing, treating, and often reversing lifestyle-related chronic diseases [1]. This approach combines six different pillars in adults: a predominantly whole-food, plant-based diet, regular physical activity, adequate sleep, stress management, healthy relationships, and avoidance of risky substances [1,2]. Eighty percent of chronic non-communicable diseases are preventable with lifestyle changes [3], including 70% of colon cancers, 70% of strokes, 80% of coronary heart diseases, and 90% of type 2 diabetes [3]. The risk factors for the leading causes of death worldwide are lifestyle-dependent [4]. However, most literature and training in lifestyle medicine has focused on adults.

Children are now presenting lifestyle-related chronic conditions at a much younger age, highlighting an urgent need for early lifestyle interventions [5]. By instilling healthy habits from a younger age, we could have a much greater impact [6,7]. The creation of pediatric lifestyle medicine is crucial because it allows for true primary prevention. It gives us the opportunity to significantly improve their quality of life, positively impact their mental health and happiness, and establish the foundations of healthy habits. A pediatric lifestyle medicine approach can even be adopted from pregnancy or pre-conception [8], impacting epigenetics [9].

**Figure 1:**
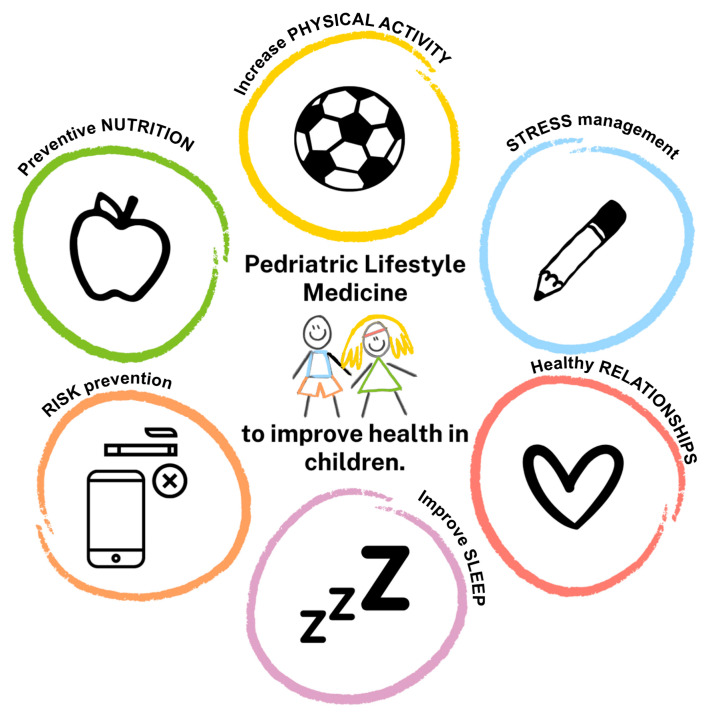
**Pediatric lifestyle medicine to improve health in children.** Pediatric lifestyle medicine is an evidence-based healthcare discipline focused on promoting and maintaining children’s health by encouraging healthy habits and behaviors from an early age. This approach aims to prevent chronic diseases from childhood and instill a lifestyle that supports physical and emotional well-being, fostering long-term quality of life.

Pediatric lifestyle medicine is defined here as an evidence-based healthcare discipline focused on promoting and maintaining children’s health by encouraging healthy habits and behaviors from an early age. This approach aims to prevent chronic diseases from childhood and instill a lifestyle that supports physical and emotional well-being, fostering long-term quality of life (Figure 1).

## 2. Methods

This study is a narrative review aimed at synthesizing the existing literature on pediatric lifestyle medicine. A comprehensive search was conducted in PubMed using both MeSH terms and free-text keywords related to the fundamental pillars of lifestyle medicine, including nutrition, physical activity, sleep, stress management, social connections, and the avoidance of risky behaviors and excessive screen time. Additionally, reference lists from relevant articles were reviewed to ensure a broad and representative selection of studies. This approach builds upon the well-established principles of adult lifestyle medicine, adapting and analyzing the available evidence specifically in pediatric populations to provide valuable insights into the role of lifestyle factors in pediatric health.

## 3. The Relevance of Pediatric Lifestyle Medicine

### 3.1. Cardiovascular Health

When considering non-communicable chronic diseases, we generally think of older adults, but studies show that these conditions start at a very early age [10]. Autopsies of young, healthy individuals who died due to accidents reveal early signs of atherosclerosis. A study of 300 autopsies of young men who died during war (mean age of 22 years) showed that 77% had clear coronary signs of atherosclerosis [11]. Another study found many autopsies with fatty streaks at 10 years of age, which were almost always present after 20 years of age [12]. Fatty streaks are the initial phase of atherosclerosis plaque that continues to develop into late adulthood. Another study indicates that the risk of atherosclerosis depends on the number of unhealthy risk factors, exacerbated if they start early in life [13].

Dyslipidemia has also increased and become quite common in the pediatric population, with a current incidence between 13% and 20% [14]. The existing evidence indicates that cardiovascular disease primary prevention should start during childhood and that general health prevention should start even before pregnancy [13].

The same trend is observed with type 2 diabetes, traditionally recognized as an adult disease but now rapidly increasing in the pediatric population, especially in Western and upper-middle-income countries [15]. The incidence of type 2 diabetes increased from 3% in 1990 to 24% (per 100,000 children) in 2023 [16,17]. It is extremely important to develop new strategies in lifestyle changes to prevent this in children.

### 3.2. Childhood Obesity

Childhood obesity is one of the main challenges in modern public health. It is associated with type 2 diabetes, cardiovascular disease, arthrosis, dyslipidemia, metabolic syndrome, hypercholesterolemia, and others [18]. Adolescents with obesity have a 90% probability of being overweight or obese at 35 years of age [19]. A current worldwide study showed that among children and adolescents, obesity increased more than four times from 1990 to 2022, while the rates of underweight fell, making obesity the most common form of malnutrition [20]. Currently, in the USA, 19.7% of children are obese [21].

Pediatrics has faced enormous public health challenges in recent decades, such as combating malnutrition and infectious diseases. However, the scenario has drastically changed, and so must our clinical and public health strategies.

A common perceived difficulty for families is that healthy food is more expensive. A study on monthly grocery shopping showed that most of the family food budget is spent on meat, bread, sugary drinks, and sweets [22]. Only 27.5% of children aged 2–12 eat legumes twice per week, 62.3% eat only one portion of fruits or vegetables per day, 30.7% eat fast food weekly, and 55.3% consume sugary drinks and juices daily [22]. They compared the consumption of these products between the lowest and highest income percentiles: legume consumption was even lower in the higher income population, sweet and junk food consumption was the same across income percentiles, and sugary beverage consumption was higher in the lower income population [22]. Another study revealed that 36% of children and adolescents in the USA consume fast food on any given day, with 11% obtaining over 45% of their total daily calories from fast food [23]. This demonstrates that education and healthy habits are essential for the entire population, irrespective of income. Additionally, improving the availability of healthy whole plant-based foods—which studies indicate are typically around 30% less expensive—could be beneficial [24].

Childhood obesity is a multifactorial problem; we currently live in an environment designed to be obesogenic [25,26]. This starts from pregnancy through epigenetics and continues during infancy, for example, early breastfeeding weaning (<4 months) and excessive protein intake during the first year of life [25]. We continue with a diet that is generally low in legumes, fruits, and vegetables and high in ultra-processed food and junk food, as well as high in carcinogens such as red meat and processed meat [25]. This is followed by a stressful environment that promotes hypercaloric food consumption and a sedentary lifestyle, high in screen usage and low in exercise. This lifestyle increases stress and mental health problems, perpetuating the cycle of obesogenic factors [25].

Childhood obesity also impacts brain health. Studies show that obese adolescents have decreased inhibitory functions [27] and hyperresponsivity to food rewards and hyporesponsivity to cognitive control circuits during food-related tasks [28]. This can affect how efficiently we control overeating and obesity in an obesogenic environment [27].

However, it is important to highlight that children should not be put on diets. We need to prescribe family lifestyle changes. Diets during childhood are associated with increased eating disorders [29]. Pediatric lifestyle interventions should focus on healthier habits and better health outcomes, not on weight. Parents should act as the frontal lobe of children before they fully develop it, helping them make good decisions. It is better to offer a child a variety of fruits (allowing for independent choices) than to choose between fruits, fries, and sugary beverages. This will later become a natural decision for them. Our main tools in pediatric lifestyle medicine are to improve family habits by incorporating more whole foods and managing the family environment.

### 3.3. Mental Health

Mental health is a crucial component of child and adolescent development, and growing research highlights its connection to lifestyle factors such as physical activity, nutrition, and sleep. Disorders like anxiety, depression, and emotional and behavioral problems have increased significantly over the past decades, affecting a considerable proportion of the pediatric population [30]. Lifestyle-based interventions, including regular physical activity, a balanced diet, and adequate sleep, have demonstrated positive effects in reducing symptoms of anxiety and depression in children and adolescents [31]. As a result, international guidelines now recommend these interventions as first-line treatments [32].

For example, a study examining the relationship between modifiable lifestyle behaviors and mental health in 6640 early adolescents found that meeting the recommendation of one hour of daily exercise was associated with approximately a 35% reduction in depression scores [33]. Similarly, sleeping 9–10 h per night was linked to a 60% lower depression score, while limiting screen time to less than one hour per day was associated with nearly a 60% reduction in depression scores [33]. Another prospective study of 3436 children aged 10 to 11 years evaluated the impact of adhering to nine lifestyle recommendations [34]. Children who met 7–9 recommendations had 56% fewer mental health consultations compared to those who met only 1–3 recommendations [34], with each additional recommendation met reducing mental health visits by 15% [34]. These interventions, as part of a comprehensive pediatric lifestyle medicine approach, play a key role in the prevention and management of mental health disorders in childhood and adolescence.

### 3.4. Asthma

Other diseases presenting during childhood that can be significantly impacted by lifestyle medicine include asthma. The prevalence of asthma has increased in recent decades, with an average of 9.5% in recent years [35,36], and there are several environmental and dietary risk factors associated with this [37]. The Western diet has been associated with an increased incidence of asthma [38,39]. A recent meta-analysis also showed an association between soft drink consumption and asthma in children [40]. Children with asthma consume higher amounts of fats and less fiber than children without asthma [38]. Higher fruit and vegetable consumption may reduce the risk of developing asthma [37]. In children with asthma, the elimination of dairy and eggs for 8 weeks improved 22% of lung function versus 0.6% in controls [41]. A Western diet has been associated with higher eosinophilia and lower forced expiratory volume [38], while a plant-based diet rich in whole grains and legumes has shown a reduction in the risk of asthma in children [37,42].

### 3.5. Constipation

Another common lifestyle health complication that has increased in children is constipation, with a current incidence of 32% [43]. Constipation has been associated with low fiber intake, low water consumption, a sedentary lifestyle, and regular consumption of fried foods [43]. Dairy intolerance, increasingly common in the world, is often associated with diarrhea but also has a significant association with constipation [44]. In an RCT involving children with constipation, replacing cow’s milk with soy milk resulted in a 68% response, with resolution of anal fissures and pain, while none of the children receiving cow’s milk had a response [44]. Children with a positive response more frequently had coexisting rhinitis, dermatitis, bronchospasm, and erythema, which can all be symptoms associated with cow’s milk intolerance [44]. A soy milk replacement can be a great alternative, as it is supplemented with calcium.

## 4. What Tools Do We Have in Pediatric Lifestyle Medicine?

Lifestyle medicine focuses on changing habits and improving health through coaching. In pediatric lifestyle medicine, coaching should always target family habits. Therefore, we need to assess several factors before starting coaching. First, it is important to have a clear reason for the consultation. Are they consulting to improve family habits, because the child is having trouble at school, or due to childhood obesity or a specific disease? The reason will guide us on which pillar to start working on. Secondly, it is also important to know who decided to seek consultation. Are the parents aligned? Is the child or adolescent involved in the motivation or reluctant to make any changes?

To better understand this, we can assess the stages of behavioral change in the family using the transtheoretical model of health. This model consists of six stages of change: precontemplation, contemplation, preparation, action, maintenance, and recurrence [45]. In the early stages of precontemplation and contemplation, the focus should be primarily on education. Practical advice becomes more relevant when individuals reach the preparation phase, where they intend to take action.

For pediatric lifestyle medicine, I propose a family transtheoretical model of health to evaluate the stage each family member is in and work on synchronizing their progress. By doing so, we can guide the family toward the action phase and subsequently concentrate on coaching. If family members are at different stages of behavior change, we can prioritize education and provide small actionable steps to those ready for change, potentially motivating the rest of the family.

The age of the child is also highly relevant. In young children, coaching is primarily directed at the parents. In adolescence, we need to involve the teenagers themselves, directing the coaching to them while ensuring support from the parents, especially if the parents are in the precontemplation or contemplation phases. Parents in these early stages might fail to support a motivated adolescent effectively without fostering an environment that supports family-wide improvement. This can also happen, for example, when an adolescent wants to adopt a plant-based diet and the parents oppose it. In such cases, coaching should focus on educating the parents and providing practical support to the adolescent. Finally, we need to be prepared to adjust our recommendations and support materials based on the child’s age.

Similar to adult lifestyle medicine, pediatric lifestyle medicine can also employ SMART goals [46]. This involves making recommendations that are Specific, Measurable, Attainable, Relevant, and Time-bound, as well as evidence-based, strategic, and tailored to the patient [46]. For example, setting a goal to incorporate three fruits into daily snacks for one month. The number of changes will depend on the stage of behavior change as well as the family’s motivations and needs. Starting with small, achievable changes often motivates families as they incorporate these changes. We can then continue to add more as family habits evolve and they feel capable of more changes. We always need to have a plan for remission and encourage families to continue their follow-ups even if they do not achieve a goal.

Finally, when making recommendations, I suggest prioritizing positive interventions over negative ones. The objective is to propose habit changes that feel positive, such as “increase the number of fruits you eat every day”, rather than negative instructions like “do not eat any sweets or junk food”. The idea of positive psychological interventions [47] is that they will likely result in a reduction in junk food and sweets without generating feelings of restriction and stress. This approach differs from conventional methodology and can improve adherence. Another important point is that restrictive diets during childhood and adolescence (often implemented through negative instructions) have been associated with an increase in eating disorders and even opposite effects regarding weight gain [48,49].

## 5. The Six Pillars in Pediatric Lifestyle Medicine Evidence and Recommendations

### 5.1. Preventive Nutrition

Preventive nutrition involves a diet that not only meets nutritional requirements but also includes foods that provide short- and long-term health benefits by reducing the risk of diseases and long-term mortality. A key distinction in this approach to pediatric nutrition is that it moves beyond simply meeting basic nutrient requirements, particularly for protein and calcium, to emphasizing the long-term health benefits of dietary choices. A meta-analysis examined the long-term impact of different food groups on overall mortality [50]. It showed that legumes, whole grains, fruits, vegetables, seeds, and fish are associated with lower mortality, while red and processed meats and sugary drinks are strongly associated with higher mortality in a dose-dependent manner [50]. Additionally, refined grains, eggs, and dairy, after approximately one serving per day, were also associated with higher mortality [50]. The WHO has also classified red and processed meats as group 1 and 2 carcinogens, respectively, indicating strong and probable evidence of carcinogenicity [51].

Mediterranean and plant-based diets have consistently been shown to reduce mortality and positively impact health [52]. The PREDIMED study demonstrated that a Mediterranean diet significantly reduces mortality from all causes and cardiovascular diseases [53], but a completely plant-based diet has been associated with an even greater reduction in mortality risk in the same population [54]. A whole-food plant-based diet has been linked to a lower incidence of ischemic heart disease, type 2 diabetes, hypertension, cancer, and obesity [55]. This is largely attributed to its lower intake of saturated fats and higher intake of nutrient-dense, fiber-rich foods such as vegetables, fruits, whole grains, legumes, soy products, nuts, and seeds, which provide essential phytochemicals and antioxidants. As a result, a plant-based diet lowers cholesterol, improves serum glucose control, and reduces chronic disease [55]. Given these benefits, several leading medical organizations, including the American College of Lifestyle Medicine, have endorsed a predominantly plant-based diet [1,56], as well as the U.S. dietary guidelines for diabetes [57], cardiovascular prevention [58], and cancer [59].

What about children? In pediatric nutrition, recommendations vary more significantly between organizations compared to adult guidelines. There is a general consensus on the importance of incorporating abundant fruits, vegetables, seeds, whole grains, and legumes into children’s diets, as well as reducing ultra-processed foods. However, guidelines differ regarding the inclusion and amounts of dairy products, white meats, and red and processed meats. For instance, some organizations advocate for the consumption of dairy and lean meats as part of a balanced diet, while others recommend limiting or avoiding red and processed meats due to associated health risks. For example, the Harvard Healthy Eating Plate promotes increasing legumes, whole grains, fruits, and vegetables while limiting red meats and avoiding processed meats [60], which aligns with recommendations from the American Heart Association’s pediatric guidelines [61]. Also, the EAT-Lancet Commission recommends a plant-forward diet rich in whole grains, legumes, fruits, vegetables, nuts, and unsaturated fats, with limited and optional animal-source foods, refined grains, added sugars, and ultra-processed foods to promote both human and planetary health [62]. Similarly, a Mediterranean diet pattern has also shown benefits in infancy [63,64,65], but smaller fish, which have lower mercury levels, are safer, particularly for pregnant women and young children [66,67].

Given the variations between guidelines across different countries, it is essential to consider these recommendations from a preventive nutrition and planetary health perspective, aiming to establish early-life dietary patterns that reduce the risk of chronic diseases later on.

**Figure 2:**
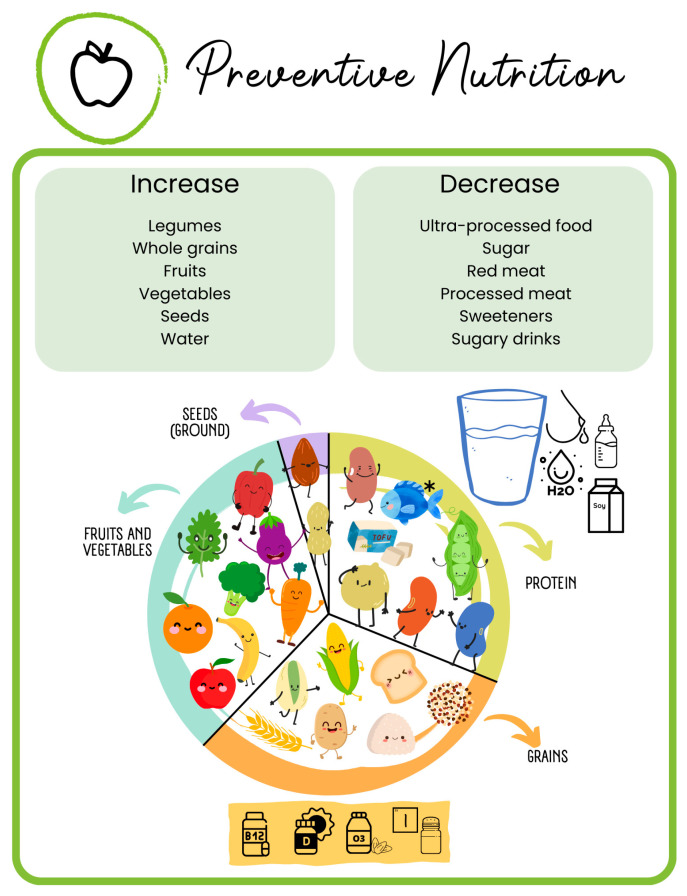
**Preventive nutrition in Pediatric Lifestyle Medicine.** * This figure illustrates the core principles of preventive nutrition in Pediatric Lifestyle Medicine, emphasizing an increase in whole plant-based foods (such as legumes, whole grains, fruits, vegetables, seeds, and water) and a reduction in ultra-processed foods, sugar, red and processed meats, sweeteners, and sugary drinks. A balanced diet should consist of approximately one-third grains (preferably whole grains, such as rice, quinoa, potatoes, and pasta), one-third protein sources (including legumes, tofu, and soy, with optional lean animal proteins such as fish or chicken), and one-third fruits and vegetables, complemented by healthy fats (such as seeds and olive oil) and adequate hydration depending on age (breast milk, water, soy milk, or low-fat cow’s milk). The risk prevention component of Pediatric Lifestyle Medicine also includes key nutrient considerations, such as supplementation of vitamin B12, vitamin D, and omega-3 fatty acids, based on individual dietary patterns. Any shift towards a more plant-based or mediterranean diet contributes to better overall health and long-term chronic disease prevention.

As we observed in adult lifestyle medicine, the primary nutrition recommendation, backed by strong scientific evidence for disease prevention and mortality reduction, is a plant-based diet. What would happen if children and adolescents adopted this diet? After reviewing the available evidence, the Academy of Nutrition and Dietetics has stated that a well-planned plant-based diet is appropriate at all stages of life, including pregnancy and childhood [55]. Plant-based diets only need to be appropriately planned and supplemented with vitamin B12 [55]. In summary, when considering preventive nutrition, pediatric lifestyle medicine recommends a shift towards increasing whole plant-based foods and decreasing sugar, ultra-processed food, and red and processed meat; any step in this direction will have a positive health impact (Figure 2) [7,8].

#### 5.1.1. What Constitutes an Appropriately Planned Diet?

A well-planned diet is essential regardless of dietary pattern, as both omnivorous and plant-based diets can lead to nutrient deficiencies if not properly structured [68]. To achieve a balance between the different nutrients, a third of the daily food intake should contain grains, preferably whole grains (such as rice, quinoa, potatoes, and pasta). Another third should include protein sources (such as legumes, tofu, and soy, with optional lean animal proteins such as fish or chicken), and the remaining third should consist of fruits and vegetables, plus some healthy fats (seeds, olive oil, etc.), accompanied by plenty of water and soy or low-fat cow’s milk (Figure 2) [55,69,70]. Any shift towards a more plant-based or mediterranean diet will contribute to improved general health and the prevention of long-term chronic diseases. To define a diet as appropriately planned, four key additional considerations should be addressed [69,71,72].

#### 5.1.2. Variety and Quantity of Whole Plant-Based Foods

The diet should include a wide variety of minimally processed whole plant-based foods. This includes diverse legumes, grains, vegetables, fruits, and seeds. Special attention should be given to fiber intake in infants starting supplementary feeding; initially, white grains, which are easier to digest, may be preferable. As the child progresses to a wider range of foods and sufficient caloric intake, there should be no restrictions on fiber.

#### 5.1.3. Careful Selection of Vegetable Fats

Prioritize sources of omega-3 fatty acids and monounsaturated oils, while avoiding trans fats and tropical oils such as coconut, palm, and palm kernel oils. In early infancy and childhood, it is important to ensure sufficient caloric intake, so vegetable fats should not be restricted. It is important to aim for sufficient omega-3 intake through the diet, primarily from seeds like chia seeds, flaxseeds, and nuts or fish. In infants under 6 months, omega-3 is obtained through breast milk, and all mothers are recommended to take omega-3 supplementation regardless of their diet. Between 6 and 12 months, introduce ½ tablespoon of flaxseed oil or ground flaxseeds or chia per day, and for children over 1 year, 1 tablespoon of ground flaxseeds or chia seeds per day is recommended. These nutrients are better absorbed when consumed in ground form or as oil [73]. If the frequency of these foods is insufficient, omega-3 supplementation should be considered, either from microalgae or fish sources.

#### 5.1.4. Vitamin B12 Supplementation

Up to a quarter of the overall population has subclinical vitamin B12 deficiency, regardless of diet [74]. Children on a primarily plant-based diet—including flexitarians, pescatarians, vegetarians, and vegans—should always receive vitamin B12 supplementation. Children on an omnivorous diet should receive it if their blood levels are low in routine controls. The dosage depends on age and B12 levels, which should be monitored to ensure they are adequate. Initially, normal B12 levels were considered to be above 211 pg/mL (156 pmol/L). However, recent studies have shown that functional deficits can still be quite frequent even at levels above this threshold [75]. To detect such deficits, measuring homocysteine levels is recommended (with normal levels being <10 μmol/L). [75] Since homocysteine testing may not always be available or may be costly, if homocysteine measurements are not feasible, B12 levels above 488 pg/mL (360 pmol/L) are now used as an alternative criterion, as this level has a 99% sensitivity for excluding functional deficiencies [76,77]. If the mother is adequately supplemented and the infant’s B12 levels are normal, supplementation should begin with 12 mcg at 6 months, and increase to 25 mcg at 4 years and 50 mcg after 8 years [69,77]. Biweekly doses are also available (Table 1). For lower levels, a loading dose may be required based on the level and age [77].

#### 5.1.5. Adequate Calcium Intake

Cow’s milk is one of many calcium sources and does not always ensure better bone health [78]. It is important to ensure sufficient calcium intake from plant-based sources such as broccoli, kale, sesame/tahini, legumes, and whole grains. Additional calcium intake can also be obtained from fortified plant-based or animal-based milks, with soy milk being the preferred plant-based choice due to its higher protein content [79]. Adequate vitamin D is also crucial, as no diet alone provides sufficient vitamin D. Supplementation should be adjusted based on geographic latitude, starting with 600–800 IU per day during infancy, and increased if there is a deficiency [69]. In the absence of lactation, modified infant formulas are safe alternatives, including soy or rice-based formulas [80].

### 5.2. Physical Activity

Physical activity is crucial for health. For adults, the recommended amount is 150 to 300 min of moderate activity per week or 75 to 150 min of vigorous activity [81]. This level of activity is associated with reduced mortality from cardiovascular disease and other causes [82].

In pediatrics, physical activity is linked to numerous health benefits, including improved cardiorespiratory and muscular function, reduced obesity, better blood pressure, lipid profiles, glucose regulation, insulin sensitivity, enhanced bone health, cognitive function, academic performance, and lower rates of depression [81,83,84,85]. Conversely, a sedentary lifestyle is associated with negative health outcomes in children and adolescents, regardless of their participation in physical activity [81]. Sedentary behavior correlates with lower well-being and quality of life [84,86], increased adiposity, higher levels of depression [87,88], and poorer laboratory parameters such as glucose, insulin, cholesterol, and triglycerides [89]. Long-term sedentary behavior also contributes to increased risks of cardiovascular diseases, diabetes, cancer, and overall mortality [90,91]. Additionally, excessive screen time combined with a sedentary lifestyle is associated with poorer sleep quality [92] and diminished social and behavioral skills [84].

Based on this evidence, current recommendations advocate for daily physical activity and the reduction in sedentary behavior [81,93]. Recent studies on strength training suggest that with proper supervision, strength exercises improve muscle strength, bone density, and academic and sports performance, reduce injury risk, and enhance motor skills and cardiovascular outcomes, without hindering growth [94,95].

**Figure 3:**
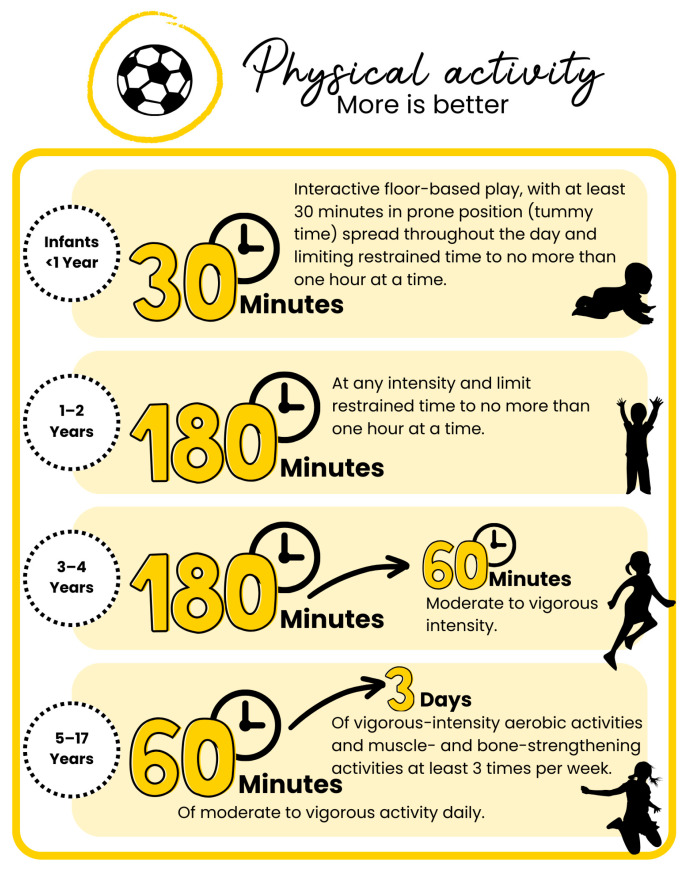
**Physical activity recommendations in Pediatric Lifestyle Medicine.** This figure outlines Pediatric Lifestyle Medicine recommendations for physical activity across different pediatric age groups. Infants (<1 year) should engage in interactive floor-based play throughout the day, including at least 30 min of prone (tummy) time while awake, with restrained time (e.g., in strollers or chairs) limited to one hour at a time. Children aged 1–2 years should accumulate at least 180 min of varied physical activity per day at any intensity while minimizing sedentary behavior and restrained time. Children aged 3–4 years should also achieve 180 min of total activity daily, including at least 60 min of moderate- to vigorous-intensity exercise daily. From age 5 to 17 years, children and adolescents should engage in at least 60 min of moderate- to vigorous-intensity activity daily, incorporating vigorous aerobic exercise and muscle- and bone-strengthening activities at least three times per week.

Pediatric lifestyle medicine should promote physical activity from infancy through adolescence. According to WHO recommendations, infants under 1 year should engage in interactive floor-based play throughout the day, including at least 30 min of prone (tummy) time while awake, and should not be restrained (e.g., in chairs or strollers) for more than an hour at a time. Children aged 1 to 2 years should have at least 3 h of varied physical activity daily, including moderate- to vigorous-intensity movement, while also minimizing sedentary time and limiting restraint in car seats or chairs to no more than one hour at a time. For children aged 3 to 4 years, these 3 h of activity should include at least 60 min of moderate- to vigorous-intensity exercise. From age 5 onward, children and adolescents should participate in at least 1 h of moderate- to vigorous-intensity activity daily, incorporating vigorous aerobic exercise and muscle- and bone-strengthening activities at least three times per week, while also reducing sedentary time (Figure 3) [81,93].

### 5.3. Sleep

Sleep is vitally important for overall health, particularly during childhood. It plays a critical role in neurodevelopment, neurocognitive and emotional health, general well-being, and quality of life [96]. Sleep also interacts with chrono-nutrition, as hormones such as melatonin, insulin, and ghrelin are regulated during sleep. For instance, late eating in children is associated with increased obesity, higher fat percentages, later breakfasts with fewer calories, and reduced sleep duration [97].

**Figure 4:**
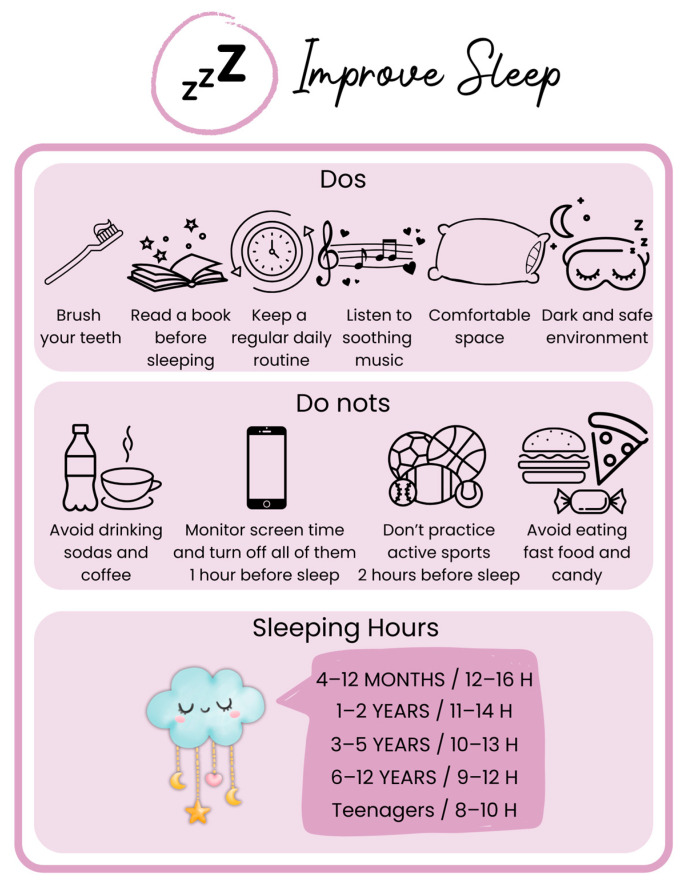
**Sleep recommendations in Pediatric Lifestyle Medicine.** Good sleep hygiene involves establishing a consistent bedtime routine, creating a dark and safe sleep environment, and ensuring a comfortable sleeping space. Positive sleep practices include brushing teeth, reading before bed, maintaining a regular daily routine, and listening to soothing music. Conversely, certain behaviors should be avoided, such as screen exposure within an hour before sleep, consuming stimulants like sodas and coffee, engaging in vigorous physical activity within two hours of bedtime, and eating large meals or sugary foods close to sleep time. Recommended sleep durations by age group are also outlined.

To promote healthy sleep, aim for adequate duration, quality, timing, and regularity [98]. Pediatric lifestyle medicine should emphasize good sleep hygiene, including a consistent routine, a dark and safe sleep environment, a comfortable sleep space, limited screen exposure, avoidance of stimulating drinks, and refraining from intense physical activity and large meals close to bedtime (Figure 4) [99,100]. Evaluations of sleep should include the total amount of sleep and daytime naps, which should be phased out after 4–5 years of age [98].

### 5.4. Stress Management

Stress management is essential for quality of life and mental health, particularly in light of pediatric mortality rates. Suicide is one of the leading causes of death among children and adolescents worldwide [101,102]. Effective strategies for improving pediatric mental health need to consider relevant risk factors such as exposure to violence, lack of social and family support, socioeconomic difficulties, and academic stress [103]. A multidisciplinary approach and access to various mental health professionals are crucial.

**Figure 5:**
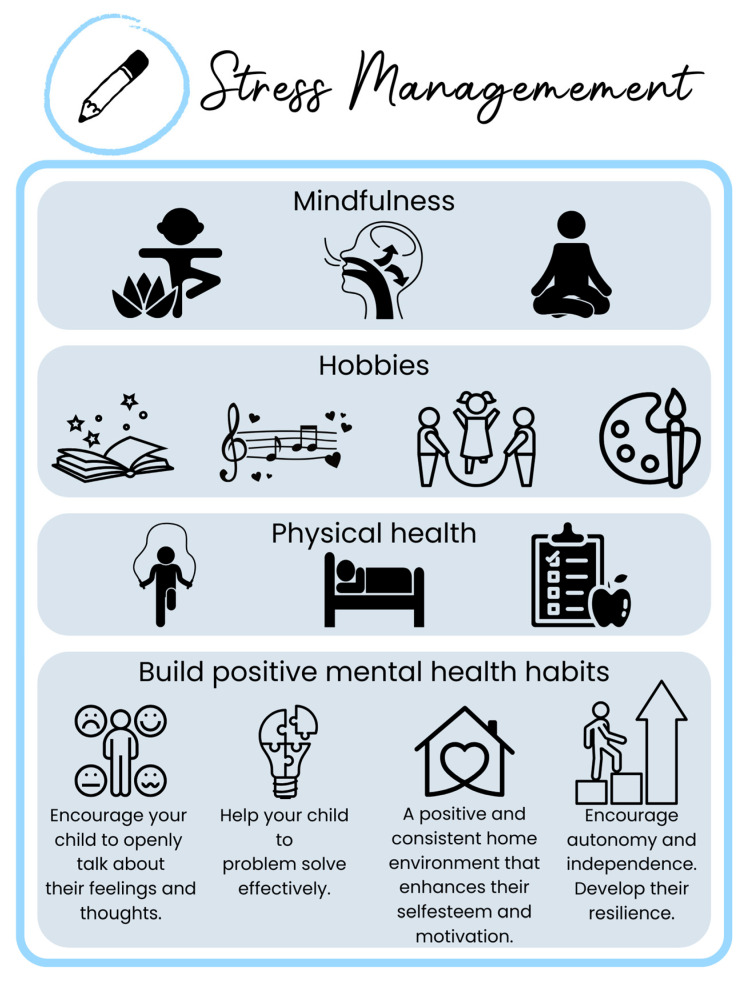
**Stress management in Pediatric Lifestyle Medicine.** Effective strategies include mindfulness practices, engaging in hobbies (such as reading, music, playtime, social activities, and creative arts), and prioritizing physical health through exercise, sleep, and nutrition. Additionally, fostering positive mental health habits within the family is crucial. Key practices involve encouraging open communication about feelings, teaching problem-solving skills, providing a stable and supportive home environment, and promoting autonomy and resilience. Incorporating playtime, reducing screen exposure, engaging with nature, and participating in sports further enhances emotional regulation, cognitive development, and stress reduction.

For adults, mindfulness is highly recommended. In children and adolescents, mindfulness interventions in schools have shown positive effects on cognitive performance, stress resilience, and psychological symptoms [104,105]. However, incorporating mindfulness in adolescence can be challenging, and results and methodologies may be inconsistent [106,107]. In pediatric care, alongside mindfulness, consider the importance of playtime, reducing screen time, engaging with nature, and participating in sports [105,108,109,110,111,112]. Play promotes social–emotional and cognitive development, self-regulation, better executive functions, and a more socially adaptive brain while reducing stress [113]. Pediatric lifestyle medicine should closely monitor mental health and its risk factors, implementing strategies that foster healthy habits, playtime, mindfulness (if desired), physical health, and positive mental health practices within the family (Figure 5).

Supportive and consistent parenting is also essential for children’s mental well-being and resilience [114]. Warm, responsive positive parenting fosters emotional regulation, while open communication helps children express their thoughts and develop problem-solving skills [115]. A positive home environment that includes parental emotional warmth and support enhances self-esteem and motivation, while encouraging autonomy builds confidence and independence [116,117]. A healthy attachment relationship, teaching coping strategies and allowing children to face manageable challenges strengthens resilience, helping them navigate stress more effectively [117,118,119]. Integrating these strategies into pediatric lifestyle medicine can significantly enhance both physical and emotional health.

### 5.5. Healthy Relationships

Research highlights the positive impact of strong social bonds on both physical and mental well-being. For example, the long-term Harvard study on happiness, which began in 1938, found that supportive and nurturing social relationships significantly contribute to overall happiness, life satisfaction, and physical health [120]. Additional studies have shown that strong social connections are associated with increased life expectancy [121].

In pediatrics, adverse childhood experiences (ACEs) have a profound impact on health and well-being. ACEs include neglect (emotional or physical), different forms of abuse (emotional, physical, and sexual), and household challenges (domestic violence, substance abuse, mental illness, parental divorce, incarceration, etc.) [122,123]. Multiple ACEs are associated with higher risks of mental health disorders, substance abuse, and physical health problems such as cardiovascular disease, diabetes, and liver disease [122,123]. A meta-analysis found that six out of ten adults report experiencing at least one ACE during childhood, and one in six report exposure to four or more ACEs [124]. ACE exposure correlates with increased risks of mental health issues, substance abuse, cancer, heart disease, respiratory disease, risky behaviors, and self-directed violence [123].

**Figure 6:**
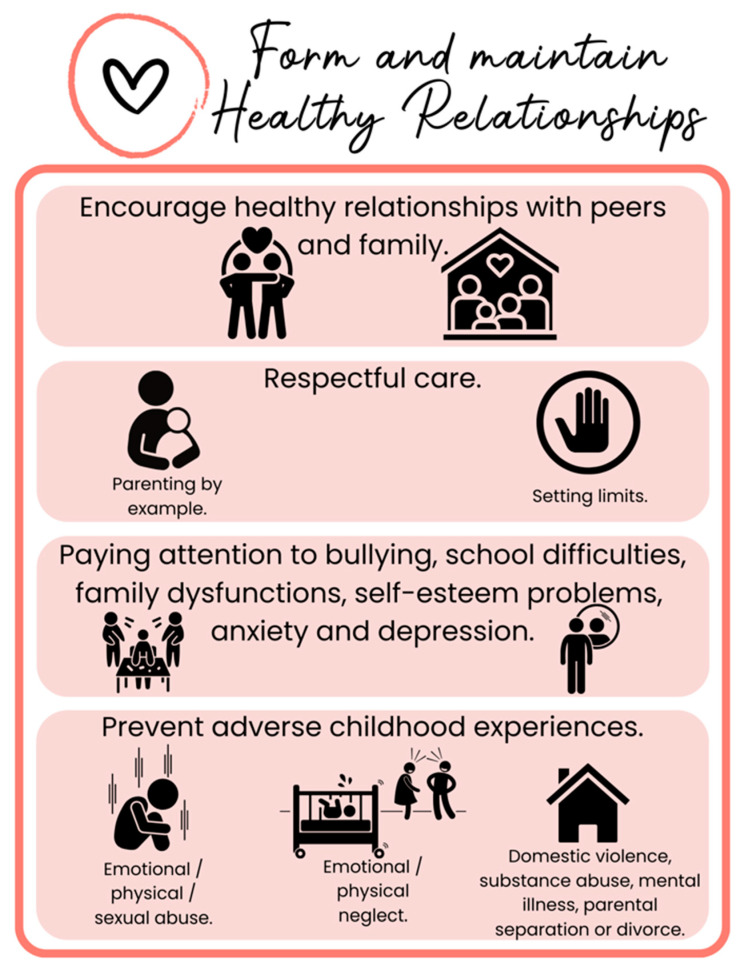
**Positive social connections in Pediatric Lifestyle Medicine.** This figure highlights key Pediatric Lifestyle Medicine strategies for fostering healthy relationships and preventing adverse childhood experiences. Encouraging positive peer and family interactions, respectful parenting, and clear boundary-setting supports emotional well-being. Monitoring bullying, school difficulties, and mental health challenges is essential. Preventing neglect, abuse, and household adversities helps reduce long-term health risks.

Proactive measures are crucial, emphasizing early interdisciplinary interventions. Efforts should focus on fostering healthy peer relationships, positive parenting practices, emotional expression, setting clear boundaries, and preventing childhood adversities and bullying incidents [125,126] (Figure 6).

### 5.6. Risk Prevention

In adult lifestyle medicine, the sixth pillar focuses on the cessation of substances such as tobacco, alcohol, and drugs, which is also crucial for adolescents [127]. However, in childhood, it can also include other relevant risk behaviors that are particularly significant in childhood, including excessive screen time, safety and accident prevention, and preventive immunization strategies. High rates of smoking, alcohol, and drug use are prevalent among adolescents, with initiation of substance use occurring at progressively younger ages, typically around 12–14 years [128,129]. Such use adversely affects brain development, leading to impaired planning, judgment, memory loss, and a lower IQ [130]. Therefore, pediatric lifestyle medicine should include regular screening for substance use, particularly during adolescence.

**Figure 7:**
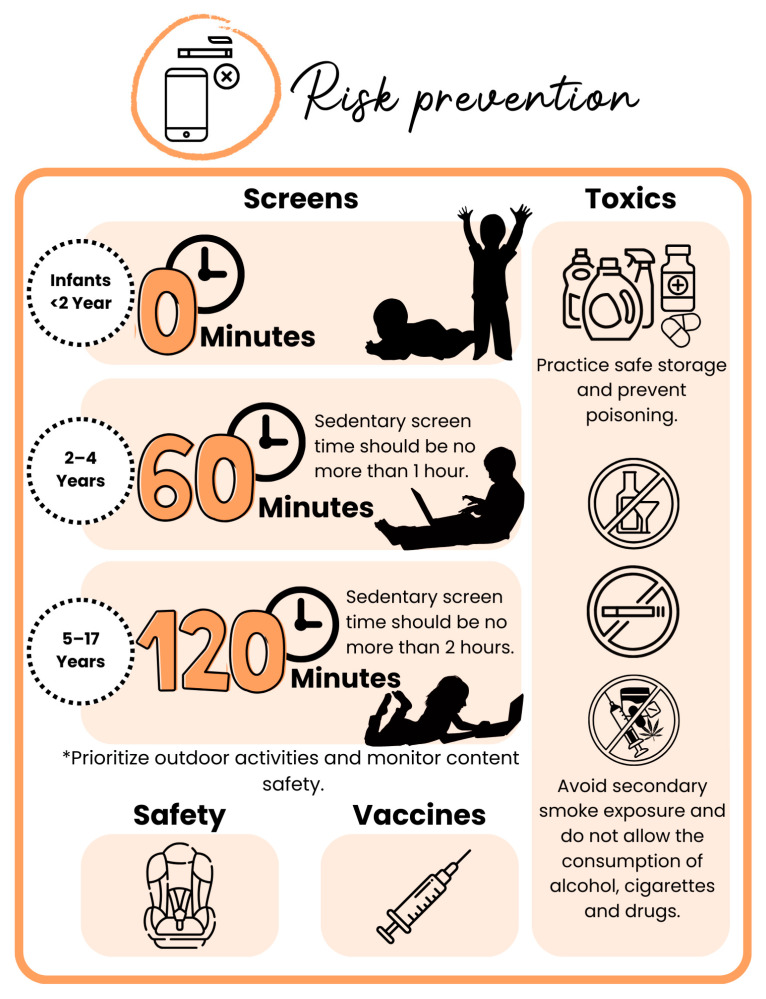
**Risk prevention in Pediatric Lifestyle Medicine.** * This pillar extends beyond substance cessation to include limiting sedentary screen time, accident prevention, safe storage of hazardous substances, and immunization strategies. Screen time recommendations suggest avoiding screens entirely for children under 2 years, limiting exposure to one hour daily for ages 2–4, and two hours daily for ages 5–17, while prioritizing outdoor activities and supervised content consumption. Toxin avoidance includes safe storage of chemicals, preventing accidental poisoning, reducing exposure to secondhand smoke, and preventing the consumption of alcohol, cigarettes and drugs. Accident prevention emphasizes the proper use of child restraint systems in vehicles and reducing exposure to violence and high-risk behaviors. Immunization remains a relevant component of disease prevention, contributing to reduced morbidity and long-term health benefits.

Another important aspect of this pillar is the prevention of accidents and accidental poisoning, significant contributors to infant mortality [131]. This includes using appropriate child restraint systems in vehicles to protect children in traffic accidents [132], which are the leading cause of death for children and youth over 4 years old in the United States [133], as well as preventing exposure to violence and injuries and sexual risk behaviors [127] (Figure 7).

In childhood, excessive screen exposure is another critical concern that can be prevented. Prolonged screen time has been associated with numerous health issues, including mental health disorders, sleep disturbances, obesity, headaches, and negative effects on neurodevelopment, attention, language acquisition, behavior, and socio-emotional and cognitive development [134,135,136]. The frontal lobe, which controls reflection, judgment, analysis, and behavior, matures later in life, around the twenties, making self-regulation more challenging for children. Screen exposure, especially to interactive and rewarding content, can activate the brain’s reward system, releasing dopamine—a neurotransmitter linked to pleasure and reward—which may reinforce the behavior and potentially lead to addiction [137,138]. Guidelines recommend avoiding screens entirely for infants under 2 years old, limiting screen time to one hour per day for toddlers aged 2–4 years, and capping it at two hours per day for children aged 5–17 years [93,136]. Recent guidelines even recommend avoiding screen exposure in children under 6 years of age a [139]. Additionally, content should be monitored, and screen use should not coincide with meal times or bedtime [93,136] (Figure 7).

Finally, within this pillar of risk prevention, childhood immunization plays a crucial role in safeguarding health and preventing disease. Vaccination not only reduces morbidity and mortality from different diseases but also contributes to better long-term health outcomes, reinforcing the importance of a comprehensive public health approach [140,141]. Integrating immunization strategies with lifestyle medicine interventions may offer a synergistic effect, reducing the burden of both infectious and non-communicable diseases in childhood.

## 6. Pediatric Lifestyle Medicine in Everyday Practice

Pediatric Lifestyle Medicine has the potential to significantly improve health and quality of life. In a context where chronic diseases are diminishing lifespan and resources are primarily invested in complex treatments, pediatric lifestyle medicine offers a cost-effective alternative for the prevention and reversal of these conditions. Additionally, with the ongoing climate crisis impacting the health of future generations, there is a responsibility to prioritize planetary health. The EAT-Lancet Commission concluded that a more plant-based diet could prevent 19–24% of deaths while also enhancing planetary health [62].

These pillars can be effectively incorporated into everyday pediatric clinical practice. Understanding the evidence and importance of these pillars empowers us to prioritize interventions appropriately. Although it may not be feasible to address all areas with every family and child, adjustments can be made based on the reason for consultation and the family’s readiness to change. By proactively identifying and addressing these health pillars during routine check-ups, we can improve overall well-being and mitigate disease risks in both the short and long term. Engaging children, adolescents, and their families in setting goals for lifestyle improvements and making achievable changes is crucial.

Integrating pediatric lifestyle medicine into existing pediatric care models is essential for optimizing preventive healthcare and improving long-term health outcomes. A recent study demonstrated the feasibility of incorporating lifestyle interventions into clinical practice by including both adults and adolescents over 14 years old, highlighting the potential for broader implementation across age groups [142]. Future efforts should focus on developing interdisciplinary frameworks that ensure accessibility and sustainability within different healthcare settings, adapting strategies to effectively reach younger pediatric populations. However, this can also face several challenges. A major barrier is the lack of formal training among healthcare providers, which limits their ability to effectively counsel families on lifestyle interventions. To address this, medical education programs should incorporate lifestyle medicine curricula, ensuring that pediatricians and other healthcare professionals are equipped with the necessary knowledge and skills.

Another significant challenge is time constraints in clinical practice, which often prioritize acute care over preventive strategies. Implementing structured screening tools and brief counseling models can help integrate lifestyle discussions into routine visits without significantly increasing consultation time. Additionally, referrals to multidisciplinary pediatric lifestyle medicine teams functioning outside of acute care settings can provide opportunities for more in-depth counseling, including group and family consultations. It is also essential to consider the long-term cost-effectiveness of this approach, as preventive strategies, despite requiring more time initially, can ultimately reduce healthcare costs by decreasing the burden of chronic diseases.

Additionally, socioeconomic and cultural factors can impact families’ ability to adopt healthy behaviors. Strategies such as community-based programs, policy initiatives, and the use of digital health tools can enhance accessibility and provide ongoing support for families in diverse settings. By addressing these barriers, pediatric lifestyle medicine can be more effectively implemented, leading to improved long-term health outcomes for children.

Future research in pediatric lifestyle medicine could focus on examining the short- and long-term outcomes of lifestyle interventions, particularly as they relate to long-term health trajectories extending into adulthood. It is also crucial to explore which strategies are most effective in promoting behavior change in children and families, with a particular emphasis on utilizing coaching tools—an area where current research is primarily focused on adults. Additionally, further investigation is needed into how pediatric lifestyle factors intersect with translational science as well as the implications of socio-cultural factors for improving the implementation of lifestyle changes at public and systemic levels.

This paper reviews the fundamentals of pediatric lifestyle medicine and offers recommendations for each pillar based on pediatric evidence. Further expertise in this areas can be gained through specialized training programs, such as the first Pediatric Lifestyle Medicine Certification, which commenced in 2024 [143].

## 7. Conclusions

Pediatric lifestyle medicine is an emerging field grounded in scientific evidence, designed to prevent, manage, and address diseases in children and adolescents influenced by lifestyle habits. By employing a multidisciplinary approach and involving families, this field adapts to the evolving landscape of pediatric health, where the main causes of morbidity and mortality have shifted in recent years. Embracing this approach allows for the development of innovative strategies and tools to effectively address the current challenges in pediatric care.

## Figures and Tables

**Table 1:** Oral vitamin B12 supplementation in loading or maintenance doses according to age.

	Loading Dose				Maintenance Dose
	B12 Below 75 pmol/L (100 pg/mL)	B12 Between 75 and 150 pmol/L (100–200 pg/mL)	B12 Between 150 and 220 pmol/L (200–300 pg/mL)	B12 Between 220 and 360 pmol/L (300–480 pg/mL)	Normal Levels > 360 pmol/L (>480 pg/mL)
**Adults, pregnancy and lactation**	1000 mcg per day for 4 months	1000 mcg per day for 3 months	1000 mcg per day for 2 months	1000 mcg per day for 1 month	50 mcg per day or 1000 mcg twice per week
**6 months to 3 years**	12 mcg 3 times per day or 250 mcg once per day for 4 months	12 mcg 3 times per day or 250 mcg once per day for 3 months	12 mcg 3 times per day or 250 mcg once per day for 2 months	12 mcg 3 times per day or 250 mcg once per day for 1 month	12 mcg per day (or 250 mcg twice per week if better adherence)
**4–10 years**	500 mcg per day for 4 months	500 mcg per day for 3 months	500 mcg per day for 2 months	500 mcg per day for 1 month	24 mcg per day (or 500 mcg twice per week if better adherence)
**Over 11 years**	1000 mcg per day for 4 months	1000 mcg per day for 3 months	1000 mcg per day for 2 months	1000 mcg per day for 1 month	50 mcg per day or 1000 mcg twice per week

Table translated with permission from Marina Gaínza-Lein, Medicina del estilo de vida infantil: una propuesta ante nuevos desafíos en salud pediátrica. *Pediatría en Red 6* [77].

## Data Availability

Not applicable.

## References

[1] Guthrie G.E. (2018). What Is Lifestyle Medicine?. Am. J. Lifestyle Med..

[2] Overview—American College of Lifestyle Medicine. https://lifestylemedicine.org/overview/.

[3] Willett W.C. (2002). Balancing life-style and genomics research for disease prevention. Science.

[4] Roser M., Ritchie H., Spooner F. Burden of Disease. Our World in Data 2021. https://ourworldindata.org/burden-of-disease.

[5] Shurney D., Gustafson P.A. (2020). Lifestyle Medicine in Children. Am. J. Lifestyle Med..

[6] Benigas S. (2022). Making the Case for Lifestyle Medicine. J. Fam. Pract..

[7] Dalal M., Cazorla-Lancaster Y., Chu C.G., Agarwal N. (2022). Healthy From the Start—Lifestyle Interventions in Early Childhood. Am. J. Lifestyle Med..

[8] McHugh J., Dalal M., Agarwal N. (2020). From Preconception Care to the First Day of School: Transforming the Health of New Families With Lifestyle Medicine. Am. J. Lifestyle Med..

[9] Panera N., Mandato C., Crudele A., Bertrando S., Vajro P., Alisi A. (2022). Genetics, epigenetics and transgenerational transmission of obesity in children. Front. Endocrinol..

[10] Strong J.P., Malcom G.T., Newman W.P., Oalmann M.C. (1992). Early lesions of atherosclerosis in childhood and youth: Natural history and risk factors. J. Am. Coll. Nutr..

[11] Enos W.F., Holmes R.H., Beyer J. (1953). Coronary disease among United States soldiers killed in action in Korea; preliminary report. J. Am. Med. Assoc..

[12] Stary H.C. (1989). Evolution and progression of atherosclerotic lesions in coronary arteries of children and young adults. Arteriosclerosis.

[13] Hong Y.M. (2010). Atherosclerotic cardiovascular disease beginning in childhood. Korean Circ. J..

[14] Maldonado V., Weeks B., Cho M., Turpin D., Arevalo A. (2022). Pediatric dyslipidemia. Prog. Pediatr. Cardiol..

[15] Wu H., Patterson C.C., Zhang X., Ghani R.B.A., Magliano D.J., Boyko E.J., Ogle G.D., Luk A.O.Y. (2022). Worldwide estimates of incidence of type 2 diabetes in children and adolescents in 2021. Diabetes Res. Clin. Pract..

[16] Dabelea D., Bell R.A., D’Agostino R.B., Imperatore G., Johansen J.M., Linder B., Liu L.L., Loots B., Marcovina S., Writing Group for the SEARCH for Diabetes in Youth Study Group (2007). Incidence of diabetes in youth in the United States. JAMA.

[17] Pinhas-Hamiel O., Dolan L.M., Daniels S.R., Standiford D., Khoury P.R., Zeitler P. (1996). Increased incidence of non-insulin-dependent diabetes mellitus among adolescents. J. Pediatr..

[18] Bashir A., Doreswamy S., Narra L.R., Patel P., Guarecuco J.E., Baig A., Lahori S., Heindl S.E. (2020). Childhood Obesity as a Predictor of Coronary Artery Disease in Adults: A Literature Review. Cureus.

[19] Juonala M., Magnussen C.G., Berenson G.S., Venn A., Burns T.L., Sabin M.A., Srinivasan S.R., Daniels S.R., Davis P.H., Chen W. (2011). Childhood adiposity, adult adiposity, and cardiovascular risk factors. N. Engl. J. Med..

[20] Phelps N.H., Singleton R.K., Zhou B., Heap R.A., Mishra A., Bennett J.E., Paciorek C.J., Lhoste V.P., Carrillo-Larco R.M., Stevens G.A. (2024). NCD Risk Factor Collaboration (NCD-RisC) Worldwide trends in underweight and obesity from 1990 to 2022: A pooled analysis of 3663 population-representative studies with 222 million children, adolescents, and adults. Lancet.

[21] Stierman B., Afful J., Carroll M.D., Chen T.-C., Davy O., Fink S., Fryar C.D., Gu Q., Hales C.M., Hughes J.P. (2021). National Health Statistics Reports, Number 158, 14 June 2021.

[22] Ministry of Health of Chile (2020). Radiografía Obesidad Infantil en Chile 2020, Elige Vivir Sano a Partir de Datos ENS 2016–2017.

[23] Fryar C.D., Carroll M.D., Ahluwalia N., Ogden C.L. (2020). Fast Food Intake Among Children and Adolescents in the United States, 2015–2018. NCHS Data Brief.

[24] Springmann M., Clark M.A., Rayner M., Scarborough P., Webb P. (2021). The global and regional costs of healthy and sustainable dietary patterns: A modelling study. Lancet Planet. Health.

[25] Brambilla I., Bellanca E., Pistone C., De Filippo M., Votto M., Tondina E., Licari A., Guarracino C., Marseglia G.L. (2022). Pediatric obesity: A mini-review for pediatrician. Acta Biomed..

[26] Verduci E., Di Profio E., Fiore G., Zuccotti G. (2022). Integrated Approaches to Combatting Childhood Obesity. Ann. Nutr. Metab..

[27] Reyes S., Peirano P., Peigneux P., Lozoff B., Algarin C. (2015). Inhibitory control in otherwise healthy overweight 10-year-old children. Int. J. Obes..

[28] Sadler J.R., Thapaliya G., Ranganath K., Gabay A., Chen L., Smith K.R., Osorio R.S., Convit A., Carnell S. (2023). Paediatric obesity and metabolic syndrome associations with cognition and the brain in youth: Current evidence and future directions. Pediatr. Obes..

[29] Fuentes Prieto J., Herrero-Martín G., Montes-Martínez M., Jáuregui-Lobera I., Fuentes Prieto J., Herrero-Martín G., Montes-Martínez M., Jáuregui-Lobera I. (2020). Alimentación familiar: Influencia en el desarrollo y mantenimiento de los trastornos de la conducta alimentaria. J. Negat. No Posit. Results.

[30] Child and Adolescent Mental Health (2022). 2022 National Healthcare Quality and Disparities Report [Internet].

[31] Firth J., Siddiqi N., Koyanagi A., Siskind D., Rosenbaum S., Galletly C., Allan S., Caneo C., Carney R., Carvalho A.F. (2019). The Lancet Psychiatry Commission: A blueprint for protecting physical health in people with mental illness. Lancet Psychiatry.

[32] National Institute for Health and Care Excellence (2019). Depression in Children and Young People: Identification and Management.

[33] Smout S., Gardner L.A., Newton N., Champion K.E. (2023). Dose–response associations between modifiable lifestyle behaviours and anxiety, depression and psychological distress symptoms in early adolescence. Aust. New Zealand J. Public. Health.

[34] Loewen O.K., Maximova K., Ekwaru J.P., Faught E.L., Asbridge M., Ohinmaa A., Veugelers P.J. (2019). Lifestyle Behavior and Mental Health in Early Adolescence. Pediatrics.

[35] Moorman J.E., Akinbami L.J., Bailey C.M., Zahran H.S., King M.E., Johnson C.A., Liu X. (2012). National surveillance of asthma: United States, 2001–2010. Vital Health Stat..

[36] Trends in Asthma Prevalence, Health Care Use, and Mortality in the United States, 2001–2010—PubMed. https://pubmed.ncbi.nlm.nih.gov/22617340/.

[37] Alwarith J., Kahleova H., Crosby L., Brooks A., Brandon L., Levin S.M., Barnard N.D. (2020). The role of nutrition in asthma prevention and treatment. Nutr. Rev..

[38] Berthon B.S., Macdonald-Wicks L.K., Gibson P.G., Wood L.G. (2013). Investigation of the association between dietary intake, disease severity and airway inflammation in asthma. Respirology.

[39] Han Y.-Y., Forno E., Brehm J.M., Acosta-Pérez E., Alvarez M., Colón-Semidey A., Rivera-Soto W., Campos H., Litonjua A.A., Alcorn J.F. (2015). Diet, interleukin-17, and childhood asthma in Puerto Ricans. Ann. Allergy Asthma Immunol..

[40] Al-Zalabani A.H., Elahi I.N., Katib A., Alamri A.G., Halawani A., Alsindi N.M., Almatrafi M., Wesselius A., Stewart K.F.J. (2019). Association between Soft Drinks Consumption and Asthma: A Systematic Review and Meta-Analysis. https://bmjopen.bmj.com/content/9/10/e029046.

[41] Yusoff N.A., Hampton S.M., Dickerson J.W., Morgan J.B. (2004). The effects of exclusion of dietary egg and milk in the management of asthmatic children: A pilot study. J. R. Soc. Promot. Health.

[42] Rice J.L., Romero K.M., Galvez Davila R.M., Meza C.T., Bilderback A., Williams D.L., Breysse P.N., Bose S., Checkley W., Hansel N.N. (2015). Association Between Adherence to the Mediterranean Diet and Asthma in Peruvian Children. Lung.

[43] Andreoli C.S., Vieira-Ribeiro S.A., Fonseca P.C.A., Moreira A.V.B., Ribeiro S.M.R., de Morais M.B., Franceschini S.C.C. (2019). Eating habits, lifestyle and intestinal constipation in children aged four to seven years. Nutr. Hosp..

[44] Iacono G., Cavataio F., Montalto G., Florena A., Tumminello M., Soresi M., Notarbartolo A., Carroccio A. (1998). Intolerance of cow’s milk and chronic constipation in children. N. Engl. J. Med..

[45] Prochaska J.O., Velicer W.F. (1997). The transtheoretical model of health behavior change. Am. J. Health Promot..

[46] White N.D., Bautista V., Lenz T., Cosimano A. (2020). Using the SMART-EST Goals in Lifestyle Medicine Prescription. Am. J. Lifestyle Med..

[47] Parks A.C., Biswas-Diener R. (2013). Positive interventions: Past, present, and future. Mindfulness, Acceptance, and Positive Psychology: The Seven Foundations of Well-Being.

[48] Field A.E., Austin S.B., Taylor C.B., Malspeis S., Rosner B., Rockett H.R., Gillman M.W., Colditz G.A. (2003). Relation between dieting and weight change among preadolescents and adolescents. Pediatrics.

[49] Golden N.H., Schneider M., Wood C., Daniels S., Abrams S., Corkins M., de Ferranti S., Committee on Nutrition, Committee on Adolescence, Section on Obesity (2016). Preventing Obesity and Eating Disorders in Adolescents. Pediatrics.

[50] Schwingshackl L., Hoffmann G., Lampousi A.-M., Knüppel S., Iqbal K., Schwedhelm C., Bechthold A., Schlesinger S., Boeing H. (2017). Food groups and risk of type 2 diabetes mellitus: A systematic review and meta-analysis of prospective studies. Eur. J. Epidemiol..

[51] IARC (2018). IARC Monographs on the Evaluation of Carcinogenic Risks to Humans. Red Meat and Processed Meat.

[52] Crovetto F., Nakaki A., Arranz A., Borras R., Vellvé K., Paules C., Boutet M.L., Castro-Barquero S., Freitas T., Casas R. (2023). Effect of a Mediterranean Diet or Mindfulness-Based Stress Reduction During Pregnancy on Child Neurodevelopment: A Prespecified Analysis of the IMPACT BCN Randomized Clinical Trial. JAMA Netw. Open.

[53] Estruch R., Ros E., Salvadó J., Covas M., Corella D., Arós F., Gracia E., Gutiérrez V., Fiol M., Lapetra J. (2018). Primary Prevention of Cardiovascular Disease with a Mediterranean Diet Supplemented with Extra-Virgin Olive Oil or Nuts. N. Engl. J. Med..

[54] Martínez-González M.A., Sánchez-Tainta A., Corella D., Salas-Salvadó J., Ros E., Arós F., Gómez-Gracia E., Fiol M., Lamuela-Raventós R.M., Schröder H. (2014). A provegetarian food pattern and reduction in total mortality in the Prevención con Dieta Mediterránea (PREDIMED) study1234. Am. J. Clin. Nutr..

[55] Melina V., Craig W., Levin S. (2016). Position of the Academy of Nutrition and Dietetics: Vegetarian Diets. J. Acad. Nutr. Diet..

[56] Bodai B.I., Nakata T.E., Wong W.T., Clark D.R., Lawenda S., Tsou C., Liu R., Shiue L., Cooper N., Rehbein M. (2018). Lifestyle Medicine: A Brief Review of Its Dramatic Impact on Health and Survival. Perm. J..

[57] Samson S.L., Vellanki P., Blonde L., Christofides E.A., Galindo R.J., Hirsch I.B., Isaacs S.D., Izuora K.E., Wang C.C.L., Twining C.L. (2023). American Association of Clinical Endocrinology Consensus Statement: Comprehensive Type 2 Diabetes Management Algorithm—2023 Update. Endocr. Pract..

[58] 2019 ACC/AHA Guideline on the Primary Prevention of Cardiovascular Disease: A Report of the American College of Cardiology/American Heart Association Task Force on Clinical Practice Guidelines | Circulation. https://www.ahajournals.org/doi/10.1161/CIR.0000000000000678.

[59] Rock C.L., Thomson C., Gansler T., Gapstur S.M., McCullough M.L., Patel A.V., Andrews K.S., Bandera E.V., Spees C.K., Robien K. (2020). American Cancer Society guideline for diet and physical activity for cancer prevention. CA Cancer J. Clin..

[60] Harvard T.H., Chan School of Public Health, Department of Nutrition, Boston The Nutrition Source. Kid’s Healthy Eating Plate..

[61] Gidding S.S., Dennison B.A., Birch L.L., Daniels S.R., Gilman M.W., Lichtenstein A.H., Rattay K.T., Steinberger J., Stettler N., Van Horn L. (2005). Dietary Recommendations for Children and Adolescents. Circulation.

[62] Willett W., Rockström J., Loken B., Springmann M., Lang T., Vermeulen S., Garnett T., Tilman D., DeClerck F., Wood A. (2019). Food in the Anthropocene: The EAT–Lancet Commission on healthy diets from sustainable food systems. Lancet.

[63] Masini A., Dallolio L., Sanmarchi F., Lovecchio F., Falato M., Longobucco Y., Lanari M., Sacchetti R. (2024). Adherence to the Mediterranean Diet in Children and Adolescents and Association with Multiple Outcomes: An Umbrella Review. Healthcare.

[64] López-Gil J.F., García-Hermoso A., Sotos-Prieto M., Cavero-Redondo I., Martínez-Vizcaíno V., Kales S.N. (2023). Mediterranean Diet-Based Interventions to Improve Anthropometric and Obesity Indicators in Children and Adolescents: A Systematic Review with Meta-Analysis of Randomized Controlled Trials. Adv. Nutr..

[65] López-Gil J.F., García-Hermoso A., Martínez-González M.Á., Rodríguez-Artalejo F. (2024). Mediterranean Diet and Cardiometabolic Biomarkers in Children and Adolescents: A Systematic Review and Meta-Analysis. JAMA Netw. Open.

[66] EFSA Panel on Contaminants in the Food Chain (CONTAM) (2012). Scientific Opinion on the risk for public health related to the presence of mercury and methylmercury in food. EFSA J..

[67] Mercury Levels in Commercial Fish and Shellfish (1990–2012) FDA 2024. https://www.fda.gov/food/environmental-contaminants-food/mercury-levels-commercial-fish-and-shellfish-1990-2012.

[68] Passarelli S., Free C.M., Shepon A., Beal T., Batis C., Golden C.D. (2024). Global estimation of dietary micronutrient inadequacies: A modelling analysis. Lancet Glob. Health.

[69] Baroni L., Goggi S., Battaglino R., Berveglieri M., Fasan I., Filippin D., Griffith P., Rizzo G., Tomasini C., Tosatti M.A. (2018). Vegan Nutrition for Mothers and Children: Practical Tools for Healthcare Providers. Nutrients.

[70] Baroni L., Goggi S., Battino M. (2019). Planning Well-Balanced Vegetarian Diets in Infants, Children, and Adolescents: The VegPlate Junior. J. Acad. Nutr. Diet..

[71] Baroni L. (2015). Vegetarianism in Food-Based Dietary Guidelines. Int. J. Nutr..

[72] Agnoli C., Baroni L., Bertini I., Ciappellano S., Fabbri A., Papa M., Pellegrini N., Sbarbati R., Scarino M.L., Siani V. (2017). Position paper on vegetarian diets from the working group of the Italian Society of Human Nutrition. Nutr. Metab. Cardiovasc. Dis..

[73] Nieman D.C., Gillitt N., Jin F., Henson D.A., Kennerly K., Shanely R.A., Ore B., Su M., Schwartz S. (2012). Chia Seed Supplementation and Disease Risk Factors in Overweight Women: A Metabolomics Investigation. J. Altern. Complement. Med..

[74] Green R., Allen L.H., Bjørke-Monsen A.-L., Brito A., Guéant J.-L., Miller J.W., Molloy A.M., Nexo E., Stabler S., Toh B.-H. (2017). Vitamin B12 deficiency. Nat. Rev. Dis. Primers.

[75] Herrmann W., Geisel J. (2002). Vegetarian lifestyle and monitoring of vitamin B-12 status. Clin. Chim. Acta.

[76] Jarquin Campos A., Risch L., Nydegger U., Wiesner J., Vazquez Van Dyck M., Renz H., Stanga Z., Risch M. (2020). Diagnostic Accuracy of Holotranscobalamin, Vitamin B12, Methylmalonic Acid, and Homocysteine in Detecting B12 Deficiency in a Large, Mixed Patient Population. Dis. Markers.

[77] Gaínza-Lein M. (2024). Medicina del Estilo de Vida Infantil: Una Propuesta Ante NUEVOS Desafíos en Salud Pediátrica. Pediatría en Red 6..

[78] Willett W.C., Ludwig D.S. (2020). Milk and Health. N. Engl. J. Med..

[79] Romulo A. (2022). Nutritional Contents and Processing of Plant-Based Milk: A Review. IOP Conf. Ser. Earth Environ. Sci..

[80] Vandenplas Y., Castrellon P.G., Rivas R., Gutiérrez C.J., Garcia L.D., Jimenez J.E., Anzo A., Hegar B., Alarcon P. (2014). Safety of soya-based infant formulas in children. Br. J. Nutr..

[81] WHO Guidelines on Physical Activity and Sedentary Behaviour. https://www.who.int/publications-detail-redirect/9789240015128.

[82] Long-Term Leisure-Time Physical Activity Intensity and All-Cause and Cause-Specific Mortality: A Prospective Cohort of US Adults. https://www.ahajournals.org/doi/epub/10.1161/CIRCULATIONAHA.121.058162.

[83] U.S. Department of Health and Human Services (2018). 2018 Physical Activity Guidelines Advisory Committee Scientific Report.

[84] Poitras V.J., Gray C.E., Borghese M.M., Carson V., Chaput J.-P., Janssen I., Katzmarzyk P.T., Pate R.R., Connor Gorber S., Kho M.E. (2016). Systematic review of the relationships between objectively measured physical activity and health indicators in school-aged children and youth. Appl. Physiol. Nutr. Metab..

[85] Xue Y., Yang Y., Huang T. (2019). Effects of chronic exercise interventions on executive function among children and adolescents: A systematic review with meta-analysis. Br. J. Sports Med..

[86] Okely A.D., Ghersi D., Hesketh K.D., Santos R., Loughran S.P., Cliff D.P., Shilton T., Grant D., Jones R.A., Stanley R.M. (2017). A collaborative approach to adopting/adapting guidelines—The Australian 24-Hour Movement Guidelines for the early years (Birth to 5 years): An integration of physical activity, sedentary behavior, and sleep. BMC Public. Health.

[87] Hoare E., Milton K., Foster C., Allender S. (2016). The associations between sedentary behaviour and mental health among adolescents: A systematic review. Int. J. Behav. Nutr. Phys. Act..

[88] Suchert V., Hanewinkel R., Isensee B. (2015). Sedentary behavior and indicators of mental health in school-aged children and adolescents: A systematic review. Prev. Med..

[89] Leiva A.M., Martínez M.A., Cristi-Montero C., Salas C., Ramírez-Campillo R., Díaz Martínez X., Aguilar-Farías N., Celis-Morales C. (2017). El sedentarismo se asocia a un incremento de factores de riesgo cardiovascular y metabólicos independiente de los niveles de actividad física. Rev. Méd. Chile.

[90] Ekelund U., Brown W.J., Steene-Johannessen J., Fagerland M.W., Owen N., Powell K.E., Bauman A.E., Lee I.-M. (2019). Do the associations of sedentary behaviour with cardiovascular disease mortality and cancer mortality differ by physical activity level? A systematic review and harmonised meta-analysis of data from 850,060 participants. Br. J. Sports Med..

[91] Ekelund U., Steene-Johannessen J., Brown W.J., Fagerland M.W., Owen N., Powell K.E., Bauman A., Lee I.-M., Lancet Physical Activity Series 2 Executive Committe, Lancet Sedentary Behaviour Working Group (2016). Does physical activity attenuate, or even eliminate, the detrimental association of sitting time with mortality? A harmonised meta-analysis of data from more than 1 million men and women. Lancet.

[92] Belmon L.S., van Stralen M.M., Busch V., Harmsen I.A., Chinapaw M.J.M. (2019). What are the determinants of children’s sleep behavior? A systematic review of longitudinal studies. Sleep. Med. Rev..

[93] Guidelines on Physical Activity, Sedentary Behaviour and Sleep for Children Under 5 Years of Age. https://www.who.int/publications-detail-redirect/9789241550536.

[94] Comité Nacional de Medicina del Deporte Infantojuvenil (2018). Entrenamiento de la Fuerza en Niños y Adolescentes: Beneficios, Riesgos y Recomendaciones. Arch. Argent. Pediatr..

[95] Stricker P.R., Faigenbaum A.D., McCambridge T.M., LaBella C.R., Brooks M.A., Canty G., Diamond A.B., Hennrikus W., Logan K., Council on Sports Medicine and Fitness (2020). Resistance Training for Children and Adolescents. Pediatrics.

[96] Schlieber M., Han J. (2021). The Role of Sleep in Young Children’s Development: A Review. J. Genet. Psychol..

[97] Martínez-Lozano N., Tvarijonaviciute A., Ríos R., Barón I., Scheer F.A.J.L., Garaulet M. (2020). Late Eating Is Associated with Obesity, Inflammatory Markers and Circadian-Related Disturbances in School-Aged Children. Nutrients.

[98] Paruthi S., Brooks L.J., D’Ambrosio C., Hall W.A., Kotagal S., Lloyd R.M., Malow B.A., Maski K., Nichols C., Quan S.F. (2016). Recommended Amount of Sleep for Pediatric Populations: A Consensus Statement of the American Academy of Sleep Medicine. J. Clin. Sleep. Med..

[99] Galland B.C., Mitchell E.A. (2010). Helping children sleep. Arch. Dis. Child..

[100] Hall W.A., Nethery E. (2019). What does sleep hygiene have to offer children’s sleep problems?. Paediatr. Respir. Rev..

[101] Ministerio de Salud (2022). Informe de Mortalidad Por Suicidio En Chile: 2010–2019.

[102] (2021). Institute of Health Metrics and Evaluation. http://ihmeuw.org/5luz.

[103] Vicente B., Saldivia S., de la Barra F., Melipillán R., Valdivia M., Kohn R. (2012). Salud mental infanto-juvenil en Chile y brechas de atención sanitarias. Rev. Médica de Chile.

[104] Zenner C., Herrnleben-Kurz S., Walach H. (2014). Mindfulness-based interventions in schools—A systematic review and meta-analysis. Front. Psychol..

[105] Zoogman S., Goldberg S.B., Hoyt W.T., Miller L. (2015). Mindfulness interventions with youth: A meta-analysis. Mindfulness.

[106] Opie J.E., Vuong A., Welsh E.T., Gray R., Pearce N., Marchionda S., Mutch R., Khalil H. (2024). Outcomes of Best-Practice Guided Digital Mental Health Interventions for Youth and Young Adults with Emerging Symptoms: Part I. A Systematic Review of Socioemotional Outcomes and Recommendations. Clin. Child. Fam. Psychol. Rev..

[107] Burke C.A. (2010). Mindfulness-Based Approaches with Children and Adolescents: A Preliminary Review of Current Research in an Emergent Field. J. Child. Fam. Stud..

[108] Bratman G.N., Anderson C.B., Berman M.G., Cochran B., de Vries S., Flanders J., Folke C., Frumkin H., Gross J.J., Hartig T. (2019). Nature and mental health: An ecosystem service perspective. Sci. Adv..

[109] Twenge J.M., Campbell W.K. (2018). Associations between screen time and lower psychological well-being among children and adolescents: Evidence from a population-based study. Prev. Med. Rep..

[110] Section on Integrative Medicine (2016). Mind-Body Therapies in Children and Youth. Pediatrics.

[111] Gray P. (2011). The Decline of Play and the Rise of Psychopathology in Children and Adolescents. Am. J. Play..

[112] Whitebread D. (2017). Free play and children’s mental health. Lancet Child. Adolesc. Health.

[113] Yogman M., Garner A., Hutchinson J., Hirsh-Pasek K., Golinkoff R.M., Baum R., Gambon T., Lavin A., Committee on Psychosocial Aspects of Child and Family Health, Council on Communications and Media (2018). The Power of Play: A Pediatric Role in Enhancing Development in Young Children. Pediatrics.

[114] Katsantonis I., Symonds J.E. (2023). Population heterogeneity in developmental trajectories of internalising and externalising mental health symptoms in childhood: Differential effects of parenting styles. Epidemiol. Psychiatr. Sci..

[115] Newland L.A. (2014). Supportive family contexts: Promoting child well-being and resilience. Early Child. Dev. Care.

[116] Peng B., Hu N., Yu H., Xiao H., Luo J. (2021). Parenting Style and Adolescent Mental Health: The Chain Mediating Effects of Self-Esteem and Psychological Inflexibility. Front. Psychol..

[117] Sun Z. (2023). The Relationship between Parenting Style and Mental Health: The Mediating Role of Psychological Resilience. SHS Web of Conf..

[118] Masten A., Gewirtz A. (2006). Resilience in Development: The Importance of Early Childhood.

[119] Masten A.S., Barnes A.J. (2018). Resilience in Children: Developmental Perspectives. Children.

[120] Waldinger R.J., Schulz M.S. (2010). What’s love got to do with it? Social functioning, perceived health, and daily happiness in married octogenarians. Psychol. Aging.

[121] Holt-Lunstad J., Smith T.B., Layton J.B. (2010). Social relationships and mortality risk: A meta-analytic review. PLoS Med..

[122] Felitti V.J., Anda R.F., Nordenberg D., Williamson D.F., Spitz A.M., Edwards V., Koss M.P., Marks J.S. (1998). Relationship of childhood abuse and household dysfunction to many of the leading causes of death in adults. The Adverse Childhood Experiences (ACE) Study. Am. J. Prev. Med..

[123] Hughes K., Bellis M.A., Hardcastle K.A., Sethi D., Butchart A., Mikton C., Jones L., Dunne M.P. (2017). The effect of multiple adverse childhood experiences on health: A systematic review and meta-analysis. Lancet Public. Health.

[124] Madigan S., Deneault A., Racine N., Park J., Thiemann R., Zhu J., Dimitropoulos G., Williamson T., Fearon P., Cénat J.M. (2023). Adverse childhood experiences: A meta-analysis of prevalence and moderators among half a million adults in 206 studies. World Psychiatry.

[125] Seay A., Freysteinson W.M., McFarlane J. (2014). Positive Parenting. Nurs. Forum.

[126] Landstedt E., Persson S. (2014). Bullying, cyberbullying, and mental health in young people. Scand. J. Public. Health.

[127] Meek J.Y. (2012). Pediatric Lifestyle Medicine. Am. J. Lifestyle Med..

[128] Observatorio Chileno de Drogas (2020). Décimo Tercer Estudio Nacional de Drogas en Población Escolar de Chile, 2019 8° Básico a 4° Medio.

[129] The Office of Communications, SAMHSA, HHS (2021). Key Substance Use and Mental Health Indicators in the United States: Results from the 2021 National Survey on Drug Use and Health.

[130] Hamidullah S., Thorpe H.H.A., Frie J.A., Mccurdy R.D., Khokhar J.Y. (2020). Adolescent Substance Use and the Brain: Behavioral, Cognitive and Neuroimaging Correlates. Front. Hum. Neurosci..

[131] Sleet D.A., Ballesteros M.F., Borse N.N. (2010). A review of unintentional injuries in adolescents. Annu. Rev. Public. Health.

[132] Durbin D.R., Hoffman B.D., Council on Injury, Violence, and Poison Prevention (2018). Child Passenger Safety. Pediatrics.

[133] WISQARS (Web-Based Injury Statistics Query and Reporting System)|Injury Center|CDC. https://www.cdc.gov/injury/wisqars/index.html.

[134] Priftis N., Panagiotakos D. (2023). Screen Time and Its Health Consequences in Children and Adolescents. Children.

[135] Christakis D.A., Zimmerman F.J. (2007). Violent television viewing during preschool is associated with antisocial behavior during school age. Pediatrics.

[136] Hill D., Ameenuddin N., Chassiakos Y.R., Cross C., Hutchinson J., Levine A., Boyd R., Mendelson R., Moreno M., Council on Communications and Media (2016). Media and Young Minds. Pediatrics.

[137] Guellai B., Somogyi E., Esseily R., Chopin A. (2022). Effects of screen exposure on young children’s cognitive development: A review. Front. Psychol..

[138] Kuss D.J., Griffiths M.D. (2012). Online gaming addiction in children and adolescents: A review of empirical research. J. Behav. Addict..

[139] La AEP Actualiza sus Recomendaciones Sobre el Uso de Pantallas en la Infancia y Adolescencia. https://www.aeped.es/noticias/aep-actualiza-sus-recomendaciones-sobre-uso-pantallas-en-infancia-y-adolescencia.

[140] Orenstein W.A., Ahmed R. (2017). Simply put: Vaccination saves lives. Proc. Natl. Acad. Sci. USA.

[141] Immunization Agenda 2030: A Global Strategy to Leave No One Behind. https://www.who.int/publications/m/item/immunization-agenda-2030-a-global-strategy-to-leave-no-one-behind.

[142] Konswa A.A., Alolaiwi L., Alsakkak M., Aleissa M., Alotaibi A., Alanazi F.F., Rasheed A. (2023). Bin Experience of establishing a lifestyle medicine clinic at primary care level- challenges and lessons learnt. J. Taibah Univ. Med. Sci..

[143] Diplomado Medicina del Estilo de Vida Infantil—Sembrando Salud. https://sembrandosalud.cl/diplomado/.

